# Household Socioeconomic and Demographic Correlates of *Cryptosporidium* Seropositivity in the United States

**DOI:** 10.1371/journal.pntd.0004080

**Published:** 2015-09-14

**Authors:** Daniel J. Becker, James Oloya, Amara E. Ezeamama

**Affiliations:** 1 Odum School of Ecology, University of Georgia, Athens, Georgia, United States of America; 2 Department of Epidemiology & Biostatistics, College of Public Health, University of Georgia, Athens, Georgia, United States of America; Centers for Disease Control and Prevention, UNITED STATES

## Abstract

**Background:**

*Cryptosporidium* are parasitic protozoa that infect humans, domestic animals, and wildlife globally. In the United States, cryptosporidiosis occurs in an estimated 750,000 persons annually, and is primarily caused by either of the *Cryptosporidium parvum* genotypes 1 and 2, exposure to which occurs through ingestion of food or water contaminated with oocytes shed from infected hosts. Although most cryptosporidiosis cases are caused by genotype 1 and are of human origin, the zoonotic sources of genotype 2, such as livestock, are increasingly recognized as important for understanding human disease patterns. Social inequality could mediate patterns of human exposure and infection by placing individuals in environments where food or water contamination and livestock contact is high or through reducing the availability of educational and sanitary resources required to avoid exposure.

**Methodology/Principal Findings:**

We here analyzed data from the National Health and Nutritional Examination Survey (NHANES) between 1999 and 2000, and related seropositivity to *Cryptosporidium parvum* to correlates of social inequality at the household and individual scale. After accounting for the complex sampling design of NHANES and confounding by individual demographics and household conditions, we found impaired household food adequacy was associated with greater odds of *Cryptosporidium* seropositivity. Additionally, we identified individuals of non-white race and ethnicity and those born outside the United States as having significantly greater risk than white, domestic-born counterparts. Furthermore, we provide suggestive evidence for direct effects of family wealth on *Cryptosporidium* seropositivity, in that persons from low-income households and from families close to the poverty threshold had elevated odds of seropositivity relative to those in high-income families and in households far above the poverty line.

**Conclusions/Significance:**

These results refute assertions that cryptosporidiosis in the United States is independent of social marginalization and poverty, and carry implications for targeted public health interventions for *Cryptosporidium* infection in resource-poor groups. Future longitudinal and multilevel studies are necessary to elucidate the complex interactions between ecological factors, social inequality, and *Cryptosporidium* dynamics.

## Author Summary

We examined if and how social inequality in the United States influences seropositivity to *Cryptosporidium parvum*. By using nationwide data on parasite seropositivity, demographics, and household metrics of socioeconomic status provided through the National Health and Nutritional Examination Survey, we quantified how measures of social inequality affect the odds of parasite infection. After adjusting for the complex sampling design of NHANES and potential confounding by individual demographics and household conditions, we found household food inadequacy was associated with greater parasite seropositivity. Additionally, we found that individuals of non-white races and ethnicities and those born outside of the United States have significantly greater odds of seropositivity than white, domestic-born counterparts. Furthermore, our study suggests evidence for direct effects of family wealth on cryptosporidiosis risk, in that persons in low-income households have elevated odds of parasite seropositivity relative to those in high-income families. These results refute the claim that cryptosporidiosis in the United States in independent of poverty and social marginalization and carry implications for targeted public health interventions for this parasitic infection in resource-poor groups.

## Introduction

*Cryptosporidium* are parasitic protozoa that infect humans, domestic animals, and wildlife globally [1]. In humans, cryptosporidiosis is a major cause of global diarrheal illness, and in the United States an estimated 750000 cases occur annually [2]. Although direct mortality from *Cryptosporidium* infection is rare and often limited to immunocompromised individuals, cryptosporidiosis can cause significant morbidity that in turn can result in high healthcare expenses and losses to productivity [3–5]. Human cryptosporidiosis is primarily caused by either of the *Cryptosporidium parvum* genotypes 1 and 2, also known as *Cryptosporidium hominis* and *Cryptosporidium parvum* [6,7]. Human exposure to *Cryptosporidium parvum* occurs primarily through ingestion of food or water contaminated with oocytes shed from infected hosts [8,9]. While genotype 1 is limited to human transmission cycles [10], transmission of genotype 2 is based in livestock reservoir hosts such as cattle [11]. Although the majority of cryptosporidiosis cases are caused by genotype 1, the zoonotic capacity of genotype 2 is increasingly recognized as important for understanding human disease patterns [12–14]. For example, cryptosporidiosis cases in the United Kingdom are highest in agricultural areas that utilize cattle manure [15], and water-borne outbreaks in Ireland and the United States have been traced back to cattle [16,17]. Furthermore, exposure directly from livestock has also been observed, particularly among persons working closely with cattle such as children and farmworkers [18,19].

Despite potential linkages between livestock reservoir sources and cryptosporidiosis, we know less about how social inequality may mediate patterns of human exposure and infection. This is unfortunate, as *Cryptosporidium* is now included in the WHO Neglected Disease Initiative [20], and epidemiological evidence suggests low socioeconomic conditions may amplify human risk. A cross-sectional study in Venezuela found individuals residing in poor urban sectors and in thatched roof–style housing had higher *Cryptosporidium* prevalence [21]. In a similar setting in Brazil, household water and nearby domestic animals tested positive for *Cryptosporidium* oocysts [22]. Such work suggests living in physically impaired environments can increase exposure to contaminated water or animals harboring infection [23]. An impaired social environment could also influence patterns of human exposure, as individuals within these environments may lack resources necessary for proper sanitation or educational avoidance of transmission routes. For example, women in Kenyan agricultural communities had greater exposure to cattle and contaminated food and water owing to power hierarchies within households [24].

A major limitation of past work on social determinants of cryptosporidiosis is a focus on either the individual or household level at small spatial scales, restricting understanding interactions between scales and broader inference [25]. To simultaneously address individual- and household-scale socioeconomic drivers of cryptosporidiosis risk, we utilized the National Health and Nutrition Examination Survey (NHANES) from the United States to ask how poor physical and social conditions affect the odds of seropositivity to *Cryptosporidium parvum*. Several reviews of neglected infections in the United States have noted that cryptosporidiosis is without significant links to poverty or social marginalization [26,27], and socioeconomic factors remain absent in syntheses of risk factors for this disease in the United States [28]. Yet a prior analysis of NHANES found that Hispanics, African Americans, and women all have greater odds of *Cryptosporidium* seropositivity [29]. Incorporation of household-scale socioeconomics may therefore improve our understanding of how an impaired physical or social environment contextualizes these individual-level relationships. Furthermore, reorienting our focus on cryptosporidiosis towards socioeconomic determinants could offer tangible opportunities for public health interventions and environmental management.

## Methods

### Population and design

Our analysis used cross-sectional data from NHANES, a series of large nationally representative surveys conducted by the National Center for Health Statistics (NCHS) based on a stratified, multistage, probability cluster design. Data are collected through household interviews, standardized physical examinations, and collection of biological samples at mobile examination centers. A nationally representative sample is selected annually, but data are released in two-year cycles to protect confidentiality and increase statistical reliability. All data were obtained from NHANES between 1999 and 2000, the only two years for which *Cryptosporidium* serological testing occurred.

To ensure adequate sample size, NHANES 1999–2000 oversampled low-income persons, adolescents 12–19 years of age, persons ≥ 60 years of age, non-Hispanic blacks, and Mexican Americans. Data were weighted to represent the total civilian non-institutionalized U.S. household population and to account for oversampling and nonresponse to the household interview and physical examination. The weights were further ratio-adjusted by age, sex, and race and ethnicity to the U.S. population control estimates from the Current Population Survey adjusted for undercounts. Of the 5663 study participants aged 6 to 49 and who underwent physical examination, 4359 individuals had serum samples available for evaluation and had data available for relevant socioeconomic and demographic covariates for this study. Participants with hemophilia or recipients of chemotherapy within four weeks were excluded.

### Ethics statement

NHANES is reviewed and approved annually by the NCHS institutional review board, and informed written consent was obtained from all participants or their parents or legal guardians. All individual records were anonymized through unique respondent sequence numbers within NHANES.

### Determining seropositivity

Infection with *Cryptosporidium parvum* is accompanied by the production of parasite-specific antibody (Ig) of all major classes [30,31]. NHANES used an experimental enzyme-linked immunosorbent assay (ELISA) to measure IgG antibodies to two surface antigens to *Cryptosporidium parvum*, 17kDA and 27kDA [32,33]. In experimental human studies, IgG reactivity to these antigens peaks within 4 to 6 weeks [34]. The IgG response to 17kDA declines to near background levels by 4 to 6 months, whereas the same antibody response to 27kDA can remain elevated for at least 6 to 12 months [35,36]. Evidence from animal and human studies suggests that this antibody response requires inoculation with *Cryptosporidium parvum* oocysts and that seropositivity to both antigens develops after either asymptomatic or symptomatic infection [34,37]. Hence the IgG response to both antigen groups likely reflects recent or current *Cryptosporidium parvum* infection and not merely exposure [36,38].

To determine seropositivity to *Cryptosporidium parvum*, serum samples were tested for reactivity to both antigen groups through the ELISA methods detailed by NHANES and [32,33]. Briefly, sample absorbance was measured using a Molecular Devices UVmax kinetic microplate reader, and IgG levels were assigned a unit value based on the eight-point positive control standard curve with a four-parameter curve fit. The 1:50 dilution of the positive control was assigned a value of 6400 units; unknown samples with absorbance values above the standard curve were diluted further and reassayed. Cutoff values to determine seropositivity are not reported within NHANES; however, prior studies have used cutoffs for seropositivity to the 17kDA and 27kDA antigens as a sample absorbance greater than 86 units, exceeding the mean plus three standard deviations of the negative control, or exceeding 10% of the positive control [38–40].

NHANES reports seropositivity separately to the 17kDA and 27kDA antigen groups as binary outcomes. Because of our interest in the social determinants of *Cryptosporidium* seropositivity, which likely remain constant through the duration of both IgG antibody responses, we here report seropositivity as a positive IgG response to both 17kDA and 27kDA antigen groups. Following experimental studies, a seropositive response to both of these antigens represents a likely recent or current infection with either genotype 1 or genotype 2 of *Cryptosporidium parvum* [36,38]; however, a seropositive result does not distinguish between the distinct human or zoonotic sources of oocysts nor whether or not an individual is currently infected.

### Socioeconomic covariates and confounding

We examined three household-level indicators of social inequality available through NHANES: food adequacy, annual income, and the poverty income ratio (PIR). Food adequacy was defined as households reporting “enough and the kinds of food wanted,” “enough but not always the kinds of food wanted”, and “sometimes/often not enough to eat.” This variable was recoded as “enough,” “some,” and “not enough” to eat and could serve as a proxy for socioeconomic status, in which resource-poor households are unable to access adequate quantities and qualities of food [41,42]. To consider direct effects of financial resources on *Cryptosporidium* seropositivity, annual income was defined as total combined family income and was divided evenly into categories of less than $25,000, between $25,000 and $45,000, and greater than $45,000. The PIR was calculated within NHANES to provide a relative measure of income relative to poverty thresholds. Annual family income was divided by the poverty guidelines specific to family size and the appropriate year and state. A PIR less than one indicates a household below the poverty threshold, whereas a ratio of one or greater indicates income above that poverty threshold. We reclassified the PIR into even categories of households below the poverty threshold (PIR < 1), households one to three times above the poverty threshold (PIR 1–3), and households with income more than three times above the poverty threshold (PIR 3+).

In addition to these primary household socioeconomic variables, we also considered confounding by the source and treatment status of household water, as *Cryptosporidium parvum* is predominantly transmitted through water-borne pathways and untreated water may also be symptomatic of low socioeconomic status. The source of household water was defined as a private or public water company, a private or public well, or another source. Water treatment was defined as whether or not the following treatment devices were used within a household: pitcher water filter, ceramic or charcoal filter, water softener, aerator, or reverse osmosis. Our analysis also considered the size of a household, defined as the number of rooms per home, to account for larger households potentially stemming from greater wealth.

We also considered confounding by demographic covariates of individual race and ethnicity, age, gender, country of birth, and education [29]. Age was defined in one-year intervals, and race and ethnicity were defined by self-report and categorized as non-Hispanic white, non-Hispanic black, Mexican American, and other. Country of birth was categorized as the United States, Mexico, or other. Education was defined by the highest grade of school completed, which we reclassified into a binary variable describing whether or not individuals completed high school. Lastly, associations between socioeconomic status and *Cryptosporidium parvum* could be confounded by individual health status, as immunocompromised persons are more susceptible to infection [1,43]. Lymphocytes play key roles in the immune defense against *Cryptosporidium*, and in particular T helper lymphocytes (CD4+ cells) are required for parasite clearance [31,44]. We therefore considered the number of thousand lymphocytes per microliter of blood in our analyses, derived from the total count of leukocytes times the differential count of percent lymphocytes. Although NHANES has directly quantified CD4+ counts, these data were only available for human immunodeficiency virus–positive individuals, which represent a very small subset of the sample tested for *Cryptosporidium* seropositivity (n = 34 records, < 1% of dataset).

### Statistical analysis

All statistical procedures were conducted to account for the design of multistage stratified, cluster-sampled, unequally weighted survey samples such as NHANES using the package *survey* in R [45,46]. All seroprevalence estimates were weighted to represent the total U.S. population and to account for over-sampling and nonresponse to interviews and physical examinations [47]. We first performed univariate analyses of primary exposures and potential confounders by fitting survey-weighted generalized linear models with the binomial outcome as the serological response to the 17kDA and 27kDA antigens. These logistic regressions were fit through pseudolikelihood and used inverse-probability weighting and design-based standard errors [48,49]. Standard error estimates were calculated using the Taylor series linearization method to account for the complex sampling design [50], and we used a Wald test to test if all coefficients associated with each covariate differed from zero to examine overall variable significance [51,52]. To adjust for the NHANES survey design, the degrees of freedom for Wald tests were calculated as the number of primary sampling units (n = 27) minus the number of strata (n = 13). This method is recommended for retaining power when considering individual-level covariates within a survey-adjusted analysis [49,53]. Due to this limited degrees of freedom imposed by the sampling design (df = 14), we only included covariates with *p* < 0.20 from a Satterthwaite-adjusted *F* statistic as potential confounders to avoid overfitting final multivariable models (shown in Table 1).

**Table 1:** *Cryptosporidium parvum* IgG seropositivity to the 17kDA and 27kDA antigens among persons aged 6–49 in the United States, NHANES 1999–2000.

Variables^a^		17kDA & 27kDA antigen test			
	Sample 4359 (100%)	IgG negative 3434 (78.8%)	IgG positive 925 (21.2%)	*F*	*p*
**Food adequacy**				**3.35**	**0.07**
Enough	3190 (73.18)	2555 (74.40)	635 (68.65)		
Some	944 (21.66)	715 (20.82)	229 (24.76)		
Not enough	225 (5.16)	164 (4.78)	61 (6.59)		
**Annual income**				**2.89**	**0.09**
Less than $25,000	2051 (47.05)	1548 (45.08)	503 (54.38)		
$25,000 to $45,000	963 (22.09)	766 (22.31)	197 (21.30)		
Greater than $45,000	1345 (30.86)	1120 (32.62)	225 (24.32)		
**Poverty income ratio**				**6.47**	**0.01**
PIR < 1	1339 (30.72)	1028 (29.94)	311 (33.62)		
PIR 1–3	1799 (41.27)	1392 (40.54)	407 (44.00)		
PIR 3+	1221 (28.01)	1014 (29.53)	207 (22.38)		
**Age (years)**				**188.96**	**<0.001**
Mean (SD)	22.17 (11.95)	20.64 (11.39)	27.86 (12.26)		
**Race & ethnicity**				**81.29**	**<0.001**
White	1334 (30.60)	1147 (33.40)	187 (20.22)		
Black	1060 (24.32)	849 (24.72)	211 (22.81)		
Hispanic	1860 (42.67)	1357 (39.52)	503 (54.38)		
Other	105 (2.41)	81 (2.36)	24 (2.59)		
**Country of birth**				**68.29**	**<0.001**
United States	3470 (79.61)	2903 (84.54)	567 (61.30)		
Mexico	581 (13.33)	336 (9.78)	245 (26.49)		
Other	308 (7.07)	195 (5.68)	113 (12.22)		
**Water treatment**				**4.91**	**0.04**
Yes	855 (19.61)	700 (20.38)	155 (16.76)		
No	3504 (80.39)	2734 (79.62)	770 (83.24)		
**Education**				**4.48**	**0.05**
Less than high school	2852 (65.43)	2302 (67.04)	550 (59.46)		
High school or greater	1507 (34.57)	1132 (32.96)	375 (40.54)		
**House size (rooms)**				**2.58**	**0.13**
Mean (SD)	5.76 (1.94)	5.85 (1.96)	5.44 (1.83)		
**Gender**				2.20	0.16
Male	2123 (48.70)	1716 (49.97)	407 (44.00)		
Female	2236 (51.30)	1718 (50.03)	518 (56.00)		
**Water source**				1.42	0.28
Company	3917 (89.86)	3098 (90.22)	819 (88.54)		
Well	413 (9.47)	313 (9.11)	100 (10.81)		
Other	29 (0.67)	23 (0.67)	6 (0.65)		
**Lymphocyte number**				0.86	0.37
Mean (SD)	2.26 (0.68)	2.26 (0.69)	2.24 (0.65)		

^a^ Percentages in each row give the proportion of each variable group with a negative or positive IgG response to the 17kDA and 27kDA antigens relative to the column total. Statistics in bold show covariates where *p* < 0.15 from a Satterthwaite-adjusted *F* statistic via a Wald test with survey-adjusted degrees of freedom.

We next constructed three survey-weighted multiple logistic regression models. These models incorporated household socioeconomic variables separately owing to strong associations between each (S1 Table). For each household socioeconomic variable (food adequacy, annual income, PIR), we included confounder variables within each model to then test associations between impaired environments and *Cryptosporidium* seropositivity. We again used Wald tests as the omnibus test of covariate significance within each model, and those variables with *p* ≤ 0.05 from a Satterthwaite-adjusted *F* statistic were considered significant. We also tested for differences between groups within each exposure variable after adjusting for the potentially inflated false-discovery rate associated with multiple comparisons using the Benjamini and Hochberg correction and *multcomp* package in R [54,55]. Crude and adjusted odds ratios and 95% confidence intervals were reported for all variables in the final survey-weighted models. Odds ratios and confidence intervals from the survey-weighted logistic regression models were also calculated using the adjusted degrees of freedom described above. Owing to the cross-sectional nature of NHANES, neither cause–effect relationships nor distinction between recent or current *Cryptosporidium* infection can be established, and hence odds ratios should be interpreted accordingly.

## Results

Of the 4359 persons tested for seropositivity to the *Cryptosporidium parvum* 17kDA and 27kDA antigen groups in our NHANES sample, 925 persons were IgG positive. This corresponds to a weighted seroprevalence for individuals aged 6 and 49 years of 21.2% (95% CI = 18.5–23.9%). Seroprevalence estimates specific to household food adequacy, family income, PIR, water source and treatment, household size, age, gender, race and ethnicity, country of birth, education, and immunocompetence (lymphocyte count) are shown in Table 1. Seropositivity was influenced by all household socioeconomic variables in our univariate analyses (all *F*_2,14_ > 2.7, all *p* ≤ 0.10), with living in low-income households, close to the poverty line, and with food inadequacy associated with increased odds of seropositivity. Demographics were also strong correlates of seropositivity, with odds increasing with age (*F*_1,14_ = 188.96, *p* < 0.001); with seroprevalence higher among non-Hispanic blacks, Mexican Americans, and other racial and ethnic groups than non-Hispanic whites (*F*_3,14_ = 81.29, *p* < 0.001); and with seroprevalence higher among persons born outside the United States compared to those born within the country (*F*_2,14_ = 52.53, *p* < 0.001). Odds of seropositivity differed little between men and women (*F*_1,14_ *=* 2.2, *p* = 0.162) and were not influenced by individual lymphocyte count (*F*_1,14_ *=* 0.86, *p* = 0.371). Lastly, we found significant associations between serostatus and home water treatment (*F*_1,14_ = 4.91, *p* = 0.045) but not with the source of household water (*F*_2,14_ *=* 1.42, *p =* 0.28); household size was also a weak negative predictor of seroprevalence (*F*_1,14_ *=* 2.58, *p =* 0.132).

For multivariable models of household socioeconomic conditions and *Cryptosporidium* seropositivity, we therefore included individual age, reported race and ethnicity, country of birth, education, gender, household size, and household water treatment status as confounders. While univariate tests found no association between individual immunocompetence and *Cryptosporidium* seropositivity, we also included the total lymphocyte count in our multivariable models to adjust for immunosuppressed individuals being more susceptible to the parasite [31,44]. Additionally, although the effect of household socioeconomic status on *Cryptosporidium* seropositivity could depend on individual age and warrant age-stratified analyses, we found no support for an interaction between age and all three socioeconomic conditions (S2 Table, S1 Fig) and thus retained age as a separate fixed effect in all models.

**Fig 1:**
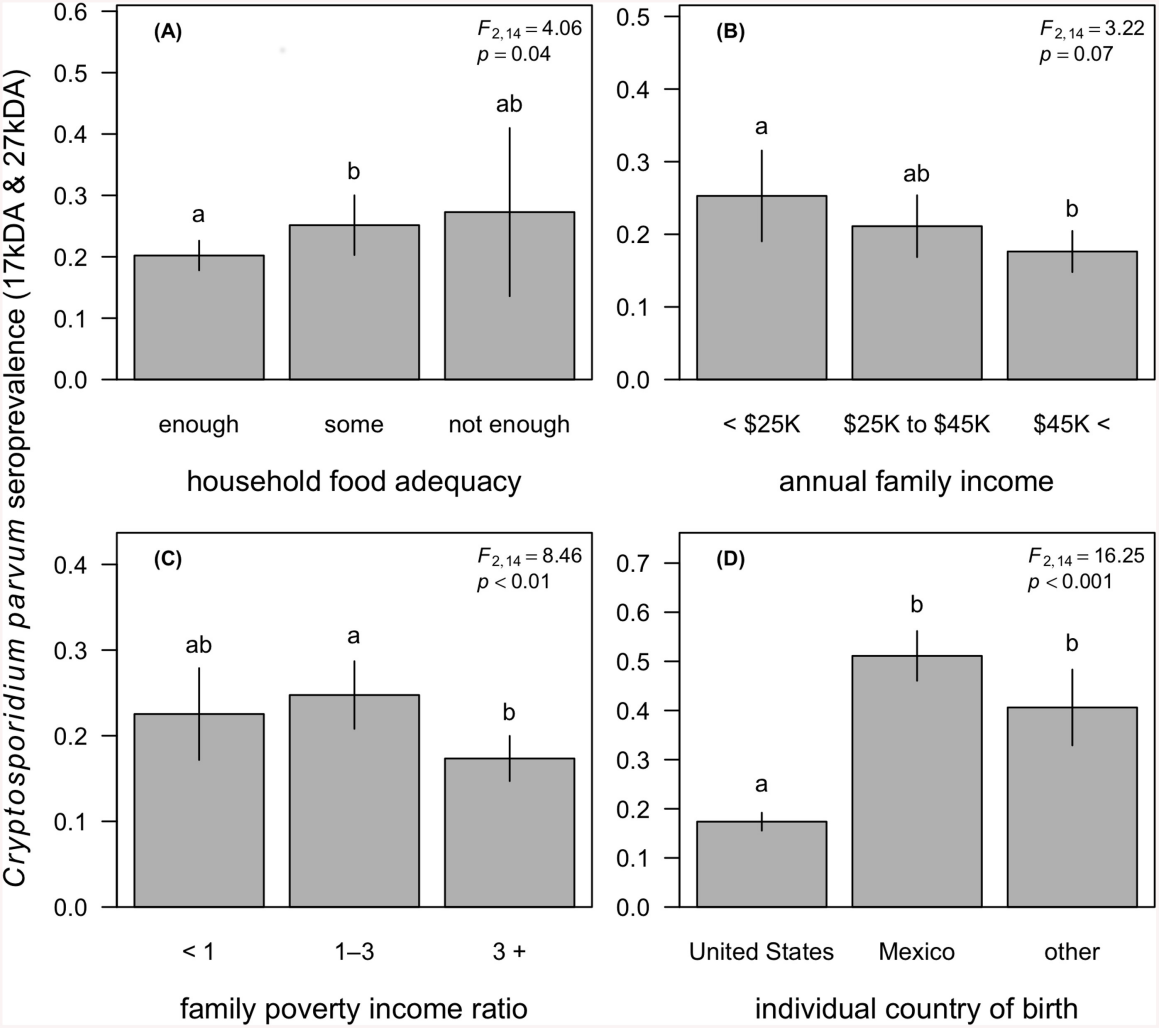
*Cryptosporidium parvum* seroprevalence (IgG response to 17kDA and 27kDA antigens) as a function of household food adequacy (A), annual family income (B), the family poverty income ratio (C), and the individual country of birth (D). Segments denote 95% confidence intervals, and letters denote significant differences between groups after adjustment for confounding and multiple comparisons. Legends report the Satterthwaite-adjusted *F* statistic and *p*-value for the primary exposure variable after adjusting for confounding, as listed in the survey-weighted models provided in Table 2.

Our survey-weighted models showed that household socioeconomic conditions were significant correlates of *Cryptosporidium* seropositivity after adjusting for individual age, race and ethnicity, country of birth, education, gender, immunocompetence, and household size and water treatment status (Table 2). We first found that household food inadequacy was associated with elevated odds of *Cryptosporidium* seropositivity (*F*_2,14_ = 4.06, *p* = 0.04; Fig 1A). After adjustment, the odds of *Cryptosporidium* seropositivity were 1.4 times higher for persons in households with some food inadequacy (OR = 1.4, *p* = 0.04) compared to those in food-adequate households. This trend appeared to be as strong for persons living in households with high food inadequacy, but this elevated odds of seropositivity compared to persons living in food-adequate households was not significant (OR = 1.31, *p* = 0.65). Likewise, we observed a negative trend between household annual income and *seropositivity*, although the overall association was not significant (*F*_2,14_ = 3.22, *p* = 0.07; Fig 1B). Compared to individuals in homes with an annual income of <$25,000 per annum, those in households with an income between $25,000 and $45,000 appeared to have lower risk, although this effect was not significant (OR = 0.78, *p* = 0.21). Yet relative to the lowest income bracket, individuals in households earning greater than $45,000 had 39% lower odds of *Cryptosporidium* seropositivity (OR = 0.61, *p* = 0.03). After adjustment for confounders, the PIR was the strongest household socioeconomic correlate of *Cryptosporidium* seropositivity and showed a non-linear relationship with seroprevalence (*F*_2,14_ = 8.46, *p* < 0.01). Relative to individuals in families living below the poverty threshold, those in households with an income one-to-three times above the poverty line had slightly elevated odds of seropositivity (OR = 1.26, *p* = 0.13), yet individuals in families with an income more than three times above than the poverty line had a suggested 25% reduction in risk (OR = 0.75, *p* = 0.14); however, neither of these differences was statistically significant. When comparing individuals in families with an annual income one-to-three times above the poverty threshold to those in families earning greater than three times the poverty line, however, this increase in income was protective against *Cryptosporidium* seropositivity (OR = 0.60, *p* < 0.001; Fig 1C).

**Table 2:** Risk factors for *Cryptosporidium parvum* IgG seropositivity to the 17kDA and 27kDA antigens among persons aged 6–49 in the United States, NHANES 1999–2000.

	Crude			Adjusted		
Risk factors and confounders^a^	Odds ratio	95% CI	*p* ^b^	Odds ratio	95% CI	*p* ^b^
**Food adequacy**			0.07			**0.04**
Enough	Ref	Ref	Ref	Ref	Ref	Ref
Some	**1.32**	**1.04–1.69**	**0.04**	**1.40**	**1.08–1.83**	**0.04**
Not enough	1.48	0.74–2.95	0.28	1.31	0.67–2.59	0.65
**Annual family income**			0.09			0.07
Less than $25,000	Ref	Ref	Ref	Ref	Ref	Ref
$25,000 to $45,000	0.79	0.53–1.22	0.32	0.78	0.54–1.14	0.21
Greater than $45,000	**0.63**	**0.49–0.95**	**0.04**	**0.61**	**0.41–0.90**	**0.03**
**Poverty income ratio**			**0.01**			**<0.01**
PIR < 1	Ref	Ref	Ref	Ref	Ref	Ref
PIR 1–3	1.13	0.87–1.46	0.36	1.26	0.97–1.64	0.13
PIR 3+	0.72	0.48–1.08	0.13	0.75	0.51–1.10	0.14
**Age (years)**	**1.05**	**1.04–1.06**	**<0.001**	**1.06**	**1.05–1.07**	**<0.001**
**Race & ethnicity**			**<0.001**			**<0.001**
White	Ref	Ref	Ref	Ref	Ref	Ref
Black	**2.01**	**1.64–2.46**	**<0.001**	**1.88**	**1.42–2.53**	**<0.001**
Hispanic	**2.68**	**2.27–3.16**	**<0.001**	**1.76**	**1.38–2.28**	**<0.001**
Other	**2.43**	**1.39–4.24**	**<0.01**	**2.13**	**1.10–4.07**	**0.04**
**Country of birth**			**<0.001**			**<0.001**
United States	Ref	Ref	Ref	Ref	Ref	Ref
Mexico	**4.97**	**3.76–6.57**	**<0.001**	**2.96**	**1.99–4.25**	**<0.001**
Other	**3.25**	**2.33–4.54**	**<0.001**	**2.27**	**1.53–3.28**	**<0.001**
**Water treatment**			**0.04**			0.12
Yes	Ref	Ref	Ref	Ref	Ref	Ref
No	**1.31**	**1.03–1.66**	**0.04**	1.19	0.97–1.45	0.13
**Gender**			0.16			0.17
Male	Ref	Ref	Ref	Ref	Ref	Ref
Female	1.20	0.94–1.52	0.16	1.22	0.94–1.59	0.17
**Education**			**0.05**			0.24
Less than high school	Ref	Ref	Ref	Ref	Ref	Ref
High school or greater	**1.26**	**1.02–1.57**	**0.05**	0.86	0.68–1.12	0.30
**Household size**	0.94	0.87–1.01	0.13	1.02	0.98–1.06	0.49
**Lymphocyte count**	0.94	0.82–1.07	0.37	1.01	0.91–1.17	0.85

^a^Adjusted odds ratios and confidence intervals are displayed from the multivariable survey-weighted logistic model including annual family income to have the most conservative estimates. Bold odds ratios and confidence intervals indicate significance at *p* ≤ 0.05 after adjusting for multiple comparisons and confounding.

^b^The *p* values presented for each covariate were derived from a Satterthwaite-adjusted *F* statistic via a Wald test and indicate an omnibus test for variable significance.

Our analyses also found individual demographics to be strong correlates of *Cryptosporidium* seropositivity. In our most conservative survey-weighted logistic model containing annual family income, we found that after adjustment for other covariates, risk of seropositivity increased by 6% per year increments in age (OR = 1.06, *p* < 0.001) and was at least 1.76 times higher for non-Hispanic blacks and Hispanics compared to non-Hispanic whites (*F*_3,14_ = 11.89, *p* < 0.001; Table 2). The odds of *Cryptosporidium* seropositivity were also at least 2.27 times higher for persons born outside the United States (*F*_2,14_ = 16.25, *p* < 0.001; Fig 1D). After adjusting for other covariates in this conservative model, we found no significant associations between *Cryptosporidium* seropositivity and household size, water treatment status, individual education, gender, or immunocompetence (Table 2).

## Discussion

Our analysis of NHANES 1999–2000 sought to examine if and how social inequality, at both household and individual scales, influenced the odds of *Cryptosporidium parvum* seropositivity. In low- and middle-income countries, *Cryptosporidium* infection associates explicitly with poverty and social marginalization [56,57]. However, regions of high socioeconomic status report greater risk in some high-income countries [15]. Such findings have perhaps prompted the claim that cryptosporidiosis is without significant links to social inequality in the United States and resulted in socioeconomic status being absent in discussions of risk factors for this infection [26–28]. Our results suggest that, contrary to these assertions, cryptosporidiosis risk in the United States is highest for individuals living in households with poor food adequacy; in families where income is low and close to the poverty threshold; and for older, non-white, and foreign-born persons.

First, we found food inadequacy was a strong household predictor of serological status, with persons in homes reporting some food inadequacy (enough to eat but not always of the foods desired) showing elevated odds of *Cryptosporidium* seropositivity. This result was robust to adjustment for household size, water treatment, age, race and ethnicity, education, gender, country of origin, and immunocompetence, yet after adjustment there was also no greater risk of seropositivity in homes with poor food adequacy compared to food-adequate households. As the direction of the food inadequacy effect suggested dose response, however, our finding of no difference in risk could be driven by the small sample size of food-inadequate households. More broadly, the significant association between food adequacy and cryptosporidiosis could suggest several pathways through which social inequality influences *Cryptosporidium* infection. Food inadequacy could first function as a proxy for household socioeconomic status, as food scarcity is typically driven by a broader lack of financial resources [41,42]. An effect of household food inadequacy through this poverty pathway could suggest reduced access to educational or sanitation resources that allow individuals to avoid *Cryptosporidium* transmission pathways such as contaminated water or contact with livestock reservoir hosts. For example, low levels of education and access to the media were both associated with poor hand washing practices and hence greater parasite exposure risk in Kenya [58].

Our multivariable models containing either annual family income or the household PIR both are suggestive of this poverty pathway, as a high annual income and living far above the poverty threshold were both protective against *Cryptosporidium* seropositivity. Yet a significant overall association between household financial resources and seroprevalence was only observed for the PIR, and for both variables we did not find a significant dose-response relationship after adjustment. Specifically, our analysis showed no difference in the odds of *Cryptosporidium* seropositivity between individuals in households living below the poverty line and those in households at any degree above the poverty threshold. Instead, we only found a difference in the odds of seropositivity between individuals in households close to the poverty threshold (1–3 times) and those in households with income greater than three times above the poverty line. These mixed findings for a direct effect of poverty on cryptosporidiosis could therefore suggest more immediate links between household food inadequacy and risk. Food inadequacy could first increase susceptibility to parasite infection through reductions to host nutritional status and therefore immunocompetence [59,60]. Food inadequacy could also push households towards accessing food products from avenues where food safety and sanitation may not be regulated, such as at open street markets [61].

This latter exposure-based pathway between household food inadequacy and cryptosporidiosis risk seems particularly plausible, as small vegetable markets have been identified as a source of *Cryptosporidium* in low-income regions [62]. Additionally, as the lymphocyte-based measure of immunocompetence had little effect on predicting seropositivity in univariate analyses and was incorporated into all multivariable models, the observed association in our multivariable model between household food inadequacy and cryptosporidiosis could be driven more by food-borne exposure rather than health status. Because household water quality was also included in our multivariable model in the form of water treatment, this potential exposure-based effect of household food inadequacy on *Cryptosporidium* seropositivity could also be driven more by consumption of contaminated produce rather than contaminated water.

Along with this household socioeconomic correlate of *Cryptosporidium* seropositivity, our analysis also identified older individuals, those of non-white ethnicities and races, and individuals born outside the United States as at greater risk of cryptosporidiosis. Increased odds of seropositivity with age are to be expected, given that the risk of ever being infected by *Cryptosporidium* would accumulate over time. For the other individual risk factors identified, another analysis of NHANES similarly found non-white Hispanics and blacks to have higher seroprevalence to the *Cryptosporidium* antigen groups used here [29]. Our finding of higher seropositivity in immigrants across the United States is also consistent with smaller-scale findings that children living along the Texas–Mexico border have higher seroprevalence than non-border children and that immigrants from Mexico have higher cryptosporidiosis prevalence than American-born counterparts in Los Angeles [63,64].

These results again highlight the potential links between social marginalization and *Cryptosporidium* seropositivity, as non-white, immigrant populations are more prone to experience unemployment, live in economically poor neighborhoods, and have reduced access to resources [65,66]. Such groups may therefore be more likely to lack access to sanitation or educational resources for avoiding parasite exposure and be prone to live in physically impaired or remote environments where access to clean food is limited or where contact with domestic animals is frequent. For example, many low-income immigrants in the United States find their employment in agriculture [67,68], which likely amplifies exposure to *Cryptosporidium* oocysts through contact with soil and water contained with livestock excrement [69]. The recreational use of open natural water sources in such regions may also elevate the odds of parasite exposure. These results in turn suggest that public health interventions for cryptosporidiosis in the United States could focus on improving awareness of *Cryptosporidium* exposure routes in such marginalized and resource-poor groups. Further research could also monitor the potential for water and food contamination in regions where high-risk groups reside and test if structural aspects of the physical environment amplify *Cryptosporidium* seropositivity [21].

Future research on the social epidemiology of cryptosporidiosis in the United States could also utilize multilevel analyses to tease apart the relative contribution of individual and household socioeconomic determinants of seropositivity while accounting for potential neighborhood effects [70,71]. Specifically, we did not find clear evidence of an overall dose–response relationship between individual seropositivity to *Cryptosporidium* and household financial resources (annual income or PIR), despite suggestive trends. Although this could be due to a small sample size for select groups of the study population, the structuring of NHANES could also have limited identifying a strong income effect. Associated geographic data (e.g., zip codes, census block) for NHANES are supplied as restricted access, and thus we only included household and individual correlates in these analyses. Yet a stronger effect of income could manifest spatially at the neighborhood scale, where low income level could cluster food-inadequate households and the demographic groups found to be at risk in our multivariable models [72,73].

An additional needed area of work on the social epidemiology of cryptosporidiosis in the United States is the pursuit of longitudinal rather than cross-sectional approaches. Owing to the cross-sectional design of NHANES within the two-year study period tested for *Cryptosporidium* seropositivity, we were unable to distinguish between IgG-positive individuals with *Cryptosporidium* infection and those that had only been recently infected and recovered. Longitudinal sampling across a socioeconomic gradient could help tease apart in which cases seropositivity is due to current or recent infections and how this varies by poverty and social marginalization, particularly if investigators measure IgM antibodies alongside IgG antibodies or changes in titers over time [74]. This could be particularly useful to account the positive association between age and seropositivity observed in this study and other analyses of NHANES [29], as IgG titers can increase with age owing to repeated *Cryptosporidium* infections. Together such studies would allow researchers to assess new *Cryptosporidium* infections in relation to acute exposures and relate direct infection to impaired social and physical conditions.

Likewise, our analysis of NHANES was limited by serological testing with the 17kDA and 27kDA antigen groups, which can identify recent or current infection with *Cryptosporidium parvum* but cannot distinguish between genotype 1 and genotype 2 of the parasite (*C*. *hominis* and *C*. *parvum*, respectively; [6]. Rather, genotyping methods on human fecal samples could elucidate whether observed cryptosporidiosis or *Cryptosporidium* seropositivity is due to infection with the human-origin genotype 1 or zoonotic genotype 2 [7,75]. Differentiation of human versus animal sources of infection in combination with analyses of socioeconomic risk factors could further improve our understanding of how impaired physical and social conditions interact with *Cryptosporidium* transmission. For example, a location-based study in the UK found that *Cryptosporidium hominis* cases were more frequent in urban areas of high socioeconomic status, whereas *Cryptosporidium parvum* cases (zoonotic genotype 2) were more common in rural areas where more oocysts were detected in agricultural soil, presumably from cattle [15].

Within the United States, genotyping methods could be particularly useful to elucidate how the individual- and household-scale correlates of poverty and social marginalization identified in our analyses interact with the abundance of livestock reservoir hosts and hence human infection with zoonotic *Cryptosporidium parvum*. Specifically, the density of livestock reservoir hosts such as cattle could be related to regional socioeconomic status [76,77], in turn driving greater exposure of marginalized groups to *Cryptosporidium parvum* genotype 2 in the United States. One analysis of sporadic cryptosporidiosis cases in Scotland accordingly found *Cryptosporidium* prevalence to be highest in rural regions with high livestock density [78], suggesting a neighborhood poverty influence on seropositivity. Hence ecological and multilevel analyses could test for an interactive influence of livestock density and social marginalization variables identified here (e.g., food adequacy, immigration status, race and ethnicity) on cryptosporidiosis while accounting for neighborhood income.

Our analyses here demonstrate clear associations between social marginalization, poverty, and cryptosporidiosis in the United States, thereby carrying important implications for targeted public health interventions for this infection in resource-poor groups. Alongside direct effects of *Cryptosporidium* infection on mortality in immunocompromised individuals, morbidity from cryptosporidiosis can range from subtle to severe effects quality of life that can impose serious restrictions on economic wellbeing [3,4]. As cryptosporidiosis is estimated to occur in 750000 persons across the United States annually, these effects can scale up to over $100 million per year in healthcare costs and losses to productivity [2,5]. Therefore, understanding interactions between socioeconomic and environmental conditions in combination with longitudinal and genotyping approaches will be key to guiding prevention and intervention strategies to cryptosporidiosis within the United States. Analyses in this spirit will more broadly help address the complex relationships between ecological factors, social inequality, and infectious disease risk [79,80].

## Supporting Information

S1 Checklist STROBE Checklist. (DOC)

S1 Table Association between household socioeconomic variables. (DOCX)

S2 Table Interactions between age and household socioeconomic status. (DOCX)

S1 Fig Visualization of age-stratifying the relationship between household socioeconomic variables and age definitions. (DOCX)
