# Supplementary material for: Household Socioeconomic and Demographic Correlates of Cryptosporidium Seropositivity in the United States
Source: PLoS Negl Trop Dis. 2015 Sep 14;9(9):e0004080. doi: 10.1371/journal.pntd.0004080 (PMC4569081; doi:10.1371/journal.pntd.0004080)
Supplement: S1 Table — (DOCX) [file pntd.0004080.s002.docx]

**S1 Table. Association between household socioeconomic variables**

|  | **Food adequacy** | **Annual income** | **PIR** |
| --- | --- | --- | --- |
| **Food adequacy** | NA | *X^2^*_4_ = 448, *p* < 0.001 | *X^2^*_4_ = 390, *p* < 0.001 |
| **Annual income** | *X^2^*_4_ = 448, *p* < 0.001 | NA | *X^2^*_4_ = 3693, *p* < 0.001 |
| **PIR** | *X^2^*_4_ = 390, *p* < 0.001 | *X^2^*_4_ = 3693, *p* < 0.001 | NA |

Prior to multivariable analyses, we assessed whether our household socioeconomic variables (food adequacy, annual income, PIR) showed strong associations with one another. Because each household socioeconomic variable was categorical, we quantified the strength of associations with a Pearson’s Chi-squared test adjusted by the survey design. These tests demonstrated that all household socioeconomic variables were strongly associated, and thus the effect of each on *Cryptosporidium* seropositivity was considered in a separate survey-weighted multivariable model.
