# Supplementary material for: Household Socioeconomic and Demographic Correlates of Cryptosporidium Seropositivity in the United States
Source: PLoS Negl Trop Dis. 2015 Sep 14;9(9):e0004080. doi: 10.1371/journal.pntd.0004080 (PMC4569081; doi:10.1371/journal.pntd.0004080)
Supplement: S2 Table — (DOCX) [file pntd.0004080.s003.docx]

**S2 Table. Interactions between age and household socioeconomic status.**

|  | SES*age  (continuous) | | SES*age  (<10, 10–30, 30+) | | SES*age  (<18, 18+) | |
| --- | --- | --- | --- | --- | --- | --- |
|  | *F*_2,14_ | *p* | *F*_4,14_ | *p* | *F*_2,14_ | *p* |
| Food adequacy | 1.53 | 0.25 | 1.81 | 0.18 | 0.37 | 0.70 |
| Annual income | 1.10 | 0.36 | 2.31 | 0.11 | 0.09 | 0.91 |
| PIR | 0.62 | 0.55 | 0.62 | 0.66 | 3.28 | 0.07 |

We also assessed the possibility that the impact of household socioeconomic status on *Cryptosporidium* seropositivity could vary by individual age. In each of our three multivariable models, we first included an interaction term between age and household socioeconomic condition, so that the linear form of each models was as follows:

Serological status ~ SES + age + SES*age + water treatment + race or ethnicity + country of birth + gender + education + lymphocyte count + household size

We tested the significance of the interaction term in each model using the Wald test and survey-adjusted degrees of freedom as described in the main text. We also considered three definitions of age: age as a continual covariate in one-year intervals; age classes defined as less than 10 years old, 10–30 years old, and greater than 30 years old; and age classes defined as less than 18 years old and greater than 18 years old. We found no support for an interaction between socioeconomics and age for any of our household variables or age definitions. Therefore, our models in the main text considered household socioeconomic condition and individual age to be separate fixed effects.
