# Supplementary material for: Household Socioeconomic and Demographic Correlates of Cryptosporidium Seropositivity in the United States
Source: PLoS Negl Trop Dis. 2015 Sep 14;9(9):e0004080. doi: 10.1371/journal.pntd.0004080 (PMC4569081; doi:10.1371/journal.pntd.0004080)
Supplement: S1 Fig — (DOCX) [file pntd.0004080.s004.docx]

**S1 Figure. Visualization of age-stratifying the relationship between household socioeconomic variables and age definitions.**


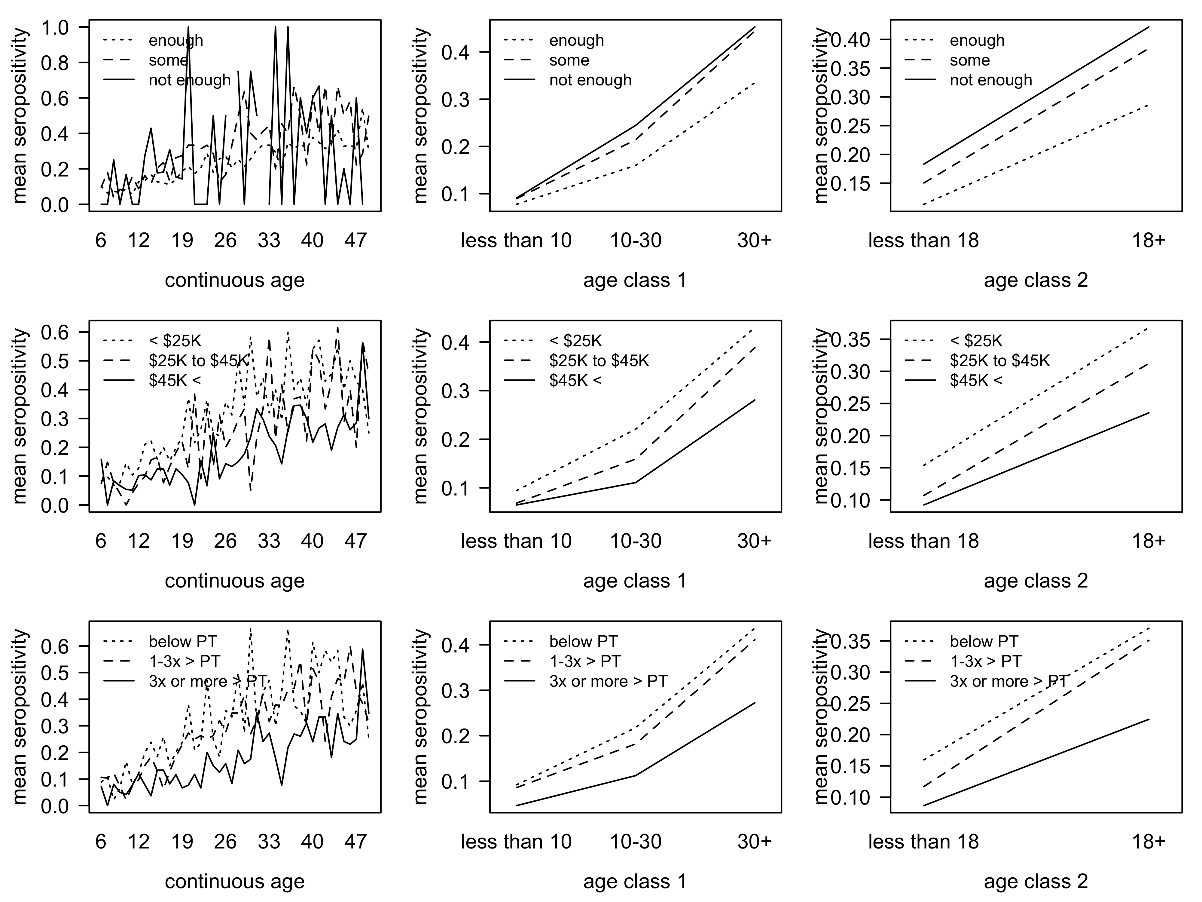


Line types represent different categories of socioeconomic status for the three household variables examined. Plots suggest a lack of age interaction across age definitions for the three household socioeconomic variables.
